# Percentage of CD56^+^ monocytes at neutrophil engraftment is associated with the incidence of acute graft-versus-host disease

**DOI:** 10.1007/s00277-026-06897-2

**Published:** 2026-03-02

**Authors:** Ken Hashimoto, Takahiko Sato, Yuichi Ishikawa, Yuki Okuhiro, Daisuke Sugiyama, He Zhang, Sachiko Ito, Yuichiro Inagaki, Kotaro Miyao, Masashi Sawa, Takanobu Morishita, Tatsunori Goto, Tetsuya Nishida, Nobuaki Fukushima, Kazutaka Ozeki, Ryo Hanajiri, Seitaro Terakura, Hiroyoshi Nishikawa, Hitoshi Kiyoi

**Affiliations:** 1 Department of Hematology and Oncology, Nagoya University Graduate School of Medicine, 65 Tsurumai, Showa-ku, Nagoya, 466-8550 Japan; 2 Department of Immunology, Nagoya University Graduate School of Medicine, Nagoya, Japan; 3 Department of Hematology and Oncology, Anjo Kosei Hospital, Anjo, Japan; 4 Department of Hematology, Japanese Red Cross Aichi Medical Center Nagoya Daiichi Hospital, Nagoya, Japan; 5 Department of Blood and Marrow Transplantation & Cellular Therapy, Fujita Health University, Toyoake, Japan; 6 Department of Hematology and Oncology, Konan Kosei Hospital, Konan, Japan; 7 Division of Cancer Immunology, Research Institute / Exploratory Oncology Research & Clinical Trial Center (EPOC), National Cancer Center, Tokyo/Chiba, Japan; 8 Division of Cancer Immune Multicellular System Regulation, Center for Cancer Immunotherapy and Immunobiology, Kyoto University Graduate School of Medicine, Kyoto, Japan; 9 Kindai University Faculty of Medicine, Osaka-sayama, Japan

**Keywords:** CD56, Monocyte, Hematopoietic stem cell transplantation, Acute graft-versus-host disease

## Abstract

Acute graft-versus-host disease (GVHD) is one of the serious complications following allogeneic hematopoietic stem cell transplantation (HSCT) that leads to non-relapse mortality. Although several biomarkers for acute GVHD have been proposed, no definitive predictive markers are clinically available. Recent studies have shown that a specific subset of peripheral blood monocytes expresses CD56 (neural cell adhesion molecule [NCAM]), particularly under inflammatory conditions; however, their role in allogeneic HSCT remains unclear. Single-cell RNA sequencing of peripheral blood mononuclear cells from patients with acute GVHD identified a unique monocyte subset characterized by an NCAM signature. Prospective multicolor flow cytometry analysis of peripheral blood samples revealed a transient increase in CD56⁺ monocytes at the time of neutrophil engraftment. Gene set enrichment analysis demonstrated pro-inflammatory transcriptome signatures of the CD56^+^ monocyte fraction. Notably, a reduced frequency of CD56⁺ monocytes was significantly associated with a lower incidence of acute GVHD, suggesting their potential as novel predictive cellular biomarkers of acute GVHD.

**Supplementary Information:**

The online version contains supplementary material available at 10.1007/s00277-026-06897-2.

## Introduction

Allogeneic hematopoietic stem cell transplantation (HSCT) is a potentially curative treatment for hematological diseases. However, acute graft-versus-host disease (GVHD) remains a serious complication and a leading cause of non-relapse mortality (NRM). Early prediction and risk stratification are crucial for initiating appropriate GVHD treatment. Although several biomarkers have been proposed and validated for diagnosing acute GVHD and predicting prognosis or treatment response, reliable clinical biomarkers remain elusive [1–3].

The Mount Sinai Acute GVHD International Consortium (MAGIC) algorithm is a representative integrated risk stratification model for newly diagnosed acute GVHD [4]. The algorithm originally demonstrated that the Ann Arbor GVHD scores computed by incorporating weighted plasma concentrations of TNFR1, REG3α, and ST2 measured at the onset of acute GVHD were effective for stratifying responses at 28 days after initial GVHD treatment and NRM at 6 months [4]. A French group validated this algorithm using the Hôpital Saint-Louis model, confirming its utility; however, they also noted that its predictive power was limited and added minimal value beyond clinical predictors such as GVHD grade, initial liver involvement, and age [5]. In terms of cellular biomarkers, a lower frequency of CD4^+^CD25^high^FOXP3^+^ regulatory T cells and increased CD30 expression in CD8^+^ T cells have been reported in association with acute GVHD, respectively [6, 7]. These markers have predominantly been studied at the onset of acute GVHD, with few reports focusing on predictive biomarkers measured prior to clinical manifestations of acute GVHD. Plasma ST2 concentration at day 14 post-transplantation has been associated with 6-month NRM, and another predictive algorithm proposed by the MAGIC consortium identified high-risk patients for NRM by computing plasma ST2 and REG3α concentrations on day 7 after allogeneic HSCT [8, 9]. These serum markers have indirect pathophysiological relevance and may be influenced by systemic changes after transplantation, emphasizing the need to identify early predictive cellular biomarkers for not only NRM but acute GVHD.

Here, we focused on the specific monocyte subset interacting via the neural cell adhesion molecule (NCAM, CD56) pathway, identified through single-cell RNA sequencing (scRNA-seq) analysis of peripheral blood from patients with acute GVHD. CD56 is a cell-surface molecule and a well-established marker of natural killer (NK) cells. In addition, a specific subset of peripheral blood monocytes also expresses CD56, particularly under inflammatory conditions, such as autoimmune diseases and malignancies [10]. Given the activated cellular immune responses in acute GVHD, the presence and clinical relevance of CD56⁺ monocytes should also be assessed; however, this subset has not been studied in the context of allogeneic HSCT or acute GVHD. In this study, CD56⁺ expression in post-engraftment monocytes was identified using prospective flow cytometry analysis, and pro-inflammatory transcriptional signatures of CD56^+^ monocytes were confirmed through transcriptome analysis. Furthermore, a transient increase in CD56⁺ monocytes at neutrophil engraftment preceded the onset of acute GVHD. Patients with a reduced frequency of CD56⁺ monocytes showed a significantly lower incidence of acute GVHD, suggesting their potential as novel predictive cellular biomarkers of acute GVHD.

## Materials and methods

### Patients

Patients who were scheduled to be treated with allogeneic HSCT were recruited between November 2021 and March 2022, and their peripheral blood cells were monitored for up to one year after the allogeneic HSCT. Patients with blast cells detected in their peripheral blood were not eligible to avoid contamination with leukemia-derived cells. Patients who underwent haploidentical transplantation were not included for this analysis due to extensive depletion of alloreactive T cells by post-transplant cyclophosphamide administration. Cases of primary engraftment failure or early death before neutrophil engraftment (*N* = 4) were excluded from the final analysis owing to an inability to assess post-transplant cellular kinetics. To ensure sufficient observation period for all patients, clinical outcomes were finalized based on data collected in July 2023. Table S1 summarizes the patient characteristics and clinical outcomes for each case. Acute GVHD was diagnosed and graded according to the guidelines of the Japanese Society for Transplantation and Cellular Therapy based on the criteria established by the 1994 Consensus Conference on Acute GVHD Grading [11, 12]. No patients died within 100 days after the transplantation, which is considered as a competing event for acute GVHD.

### Peripheral blood mononuclear cells

Blood samples were collected weekly for the first month after HSCT and monthly thereafter for up to one year post-transplant or until death, loss to follow-up, or when outpatient sampling could no longer be maintained. Peripheral blood mononuclear cells (PBMCs) were isolated using density gradient centrifugation with Ficoll-Paque (GE Healthcare, IL, USA) and used for subsequent experiments in a fresh state without cryopreservation. For microscopic evaluation, CD56^+^ monocytes were sorted using a FACSymphony S6 (BD Biosciences, NJ, USA). May–Giemsa staining was performed according to the manufacturer’s instructions (FUJIFILM Wako Chemicals, Japan), and images of cellular morphology were captured using ECLIPSE Ni-L (Nikon, Japan).

### Multicolor flow cytometry assay

Multicolor flow cytometry (FCM) staining, sorting, and analyses of fresh PBMCs were performed as previously described [13]. The antibodies and dyes used in this study are summarized in Tables S2 and S3. Stained cells were analyzed using a FACSymphony A3 (BD Biosciences) and FlowJo software version 10.10.0 (BD Biosciences). Fixable Viability Dyes (Thermo Fisher Scientific, MA, USA) were used to detect unstained live cells. Monocytes were defined as CD45^+^CD3^−^CD19^−^CD11b^+^CD14^+^ cells and NK cells as CD45^+^CD3^−^CD19^−^CD11b^−^CD14^−^CD56^+^ cells, respectively. The cutoff for CD56 expression in monocytes was determined based on the CD56 expression level in NK cells. Gating strategies for monocytes and NK cells are shown in Fig. S1.

### scRNA-seq data processing

Single-cell gene expression data were downloaded from the Gene Expression Omnibus database (accession number GSE255298) [14]. Scanpy version 1.10.2 and AnnData version 0.10.8 were used for data processing and visualization as we previous described [13]. Predicted doublet cells were removed using Scrublet [15]. Dimension reduction was conducted using principal component analysis of highly variable genes, and visualization was performed by Uniform Manifold Approximation and Projection (UMAP) after batch correction using Harmony package version 0.0.10 [16]. Unsupervised clustering was performed with the Leiden algorithm [17]. Gene Set Enrichment Analysis (GSEA) was conducted using GSEApy version 1.1.3 [18], with gene sets obtained from the Enrichr library (https://maayanlab.cloud/Enrichr). Cell types were assessed using the ‘CellMarker_2024’ gene set. Cell-to-cell communication was estimated using CellChat version 1.6.1 [19] after data style conversion from AnnData to Seurat object by zellkonverter version 1.12.1, which was obtained from the GitHub platform (https://theislab.github.io/zellkonverter/).

### Transcriptome analysis and data processing

CD56^+^ and CD56^−^ monocytes were sorted within 1 week of neutrophil engraftment from an independent cohort of four consecutive patients who underwent bone marrow or peripheral blood stem cell transplantation. Sorting was performed using a FACSymphony S6 (BD Biosciences) prior to the onset of acute GVHD, and the purity of each sorted population was confirmed for each sample by re-analyzing the sorted cells immediately after their sorting (Table S4 and S5). Total RNA was extracted from each case independently using TRIzol™ Reagent (Thermo Fisher Scientific) and RNeasy Micro Kit (QIAGEN, Germany), and sequenced using Illumina NovaSeq 6000 (Illumina, CA, USA). Raw FASTQ files were processed using fastp version 0.19.5 [20], and reads were aligned to the GRCh38 human reference using STAR software version 2.7.11b [21]. RSEM version 1.3.1 was used for the transcript quantification [22]. Differentially expressed genes were identified using DESeq2 version 1.42.1 [23]. GSEA was performed according to the instructions [24]. Reference human DNA sequences and comprehensive gene annotation files (GRCh38; Ensembl release 113) were obtained from https://ftp.ensembl.org/.

### Statistical analyses

The relationships between groups were compared using Fisher’s exact test or Mann–Whitney *U* test. Competing risk analyses were used to estimate the cumulative incidence of acute GVHD, and univariate analyses were performed using Gray’s test. The optimal cutoff value that could distinguish between the high- and low-risk groups for acute GVHD was determined using the Youden index in receiver operating characteristic (ROC) analysis. Survival analyses were performed using the time point of CD56^+^ monocyte assessment at neutrophil engraftment as the landmark. Statistical analyses were performed with GraphPad Prism 10 (GraphPad Software, MA, USA) or R version 4.5.0. All statistical tests were two-sided, and *P* values less than 0.05 were considered to indicate statistical significance.

## Results

### Monocyte gene signature and NCAM-mediated cell-to-cell interactions are identified in patients with acute GVHD

**Fig. 1:**
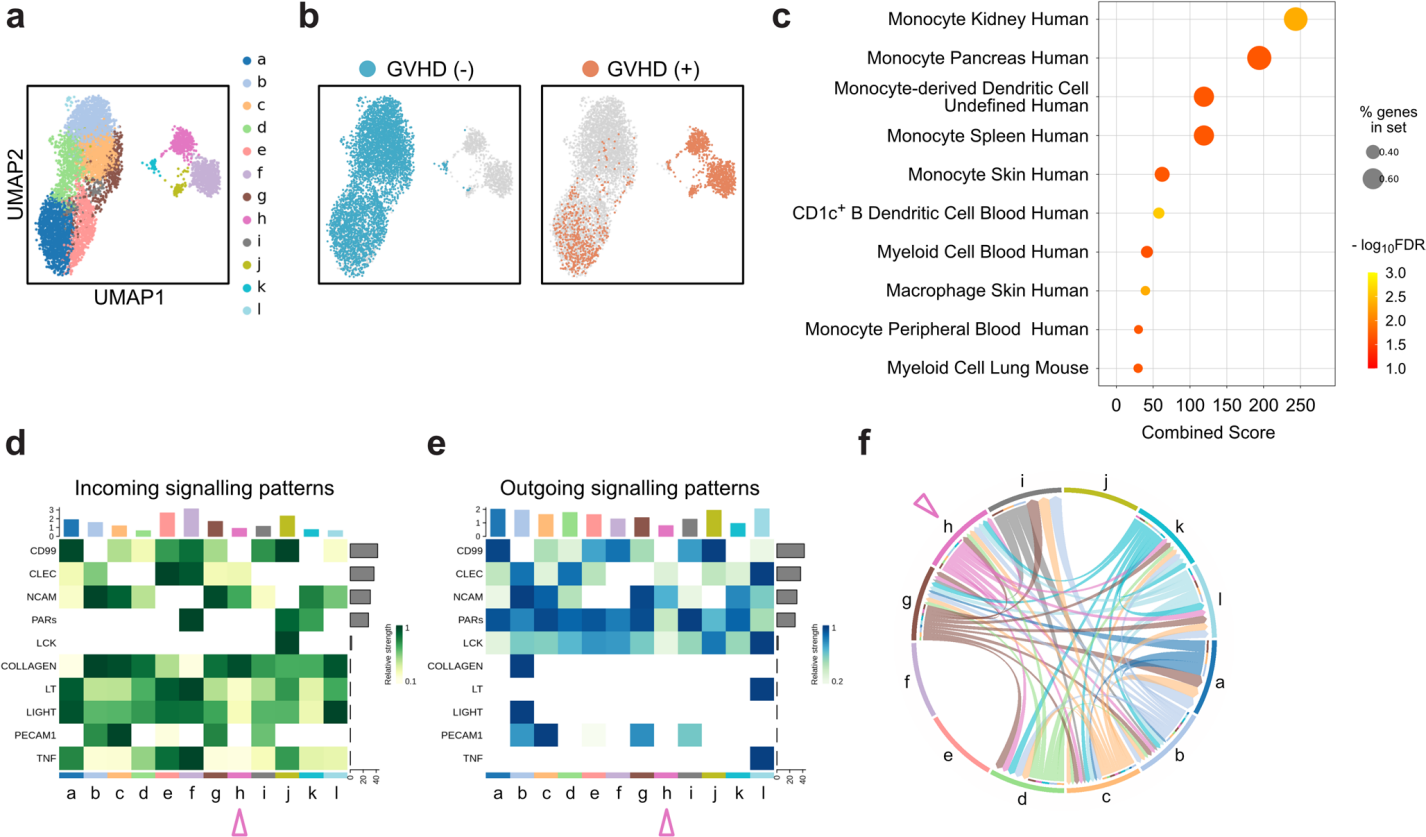
Monocyte-related transcriptional signatures and NCAM-mediated cell-to-cell interactions are enriched in patients with acute graft-versus-host disease (**a**) Uniform Manifold Approximation and Projection (UMAP) visualization of cell clusters identified by integrating the transcriptomes of peripheral blood mononuclear cells (PBMCs) collected at the time of acute graft-versus-host disease (GVHD) diagnosis and from matched controls. Twelve clusters were defined using the Leiden algorithm and are shown in different colors. (**b**) UMAP visualization based on clinical annotations according to acute GVHD. (**c**) Gene Set Enrichment Analysis (GSEA) with the ‘CellMarker_2024’ gene set library from the Enrichr was performed to compare acute GVHD samples with GVHD(-) controls. The top 10 gene sets significantly enriched in acute GVHD samples are shown as dot plots, ranked by combined scores. (**d **and **e**) Heatmaps showing predicted cell-to-cell interaction pathways estimated using the CellChat package. The cluster ‘h’, shown by the arrowheads and prevalent in cases of acute GVHD, was characterized by cell-to-cell communication via NCAM. (**f**) Chord diagram depicting NCAM signaling interactions among clusters

To investigate changes in the peripheral blood immune cells in patients with acute GVHD, a publicly available scRNA-seq dataset was examined. Batch corrections across the samples were performed using the Harmony package, followed by dimensionality reduction with UMAP. Unsupervised clustering revealed 12 distinct immune cell clusters (Fig. 1a, Fig. S2a). The integrated visualization of single cells showed that each cluster was composed of single cells from various samples (Fig. S2b and c).

UMAP plots were separately visualized for patients who developed acute GVHD and those did not, in order to identify acute GVHD-specific cellular changes. Clusters f, h, and j were particularly enriched in the GVHD group (Fig. 1b, Fig. S2d). GSEA further revealed significant enrichment of monocyte-associated gene sets in patients with acute GVHD (Fig. 1c). UMAP visualizations of the signature score showed that these monocyte-associated gene sets were enriched in clusters f and h, which were the major clusters in patients with acute GVHD (Fig. S2e). To further characterize the clusters enriched in acute GVHD cases, additional analyses were performed using the CellChat package, which predicts intercellular signaling networks (Fig. S2f). Notably, the interaction signature named NCAM was estimated specifically within cluster h (Fig. 1d–f). Taken together, these monocyte gene signatures and NCAM-mediated cell-to-cell interactions were uniquely enriched in acute GVHD-specific clusters, suggesting that the NCAM-mediated monocyte response is involved in the acute GVHD development.

### Peripheral blood analyses reveal an increased percentage of CD56^+^ monocytes following neutrophil engraftment after allogeneic HSCT

**Fig. 2:**
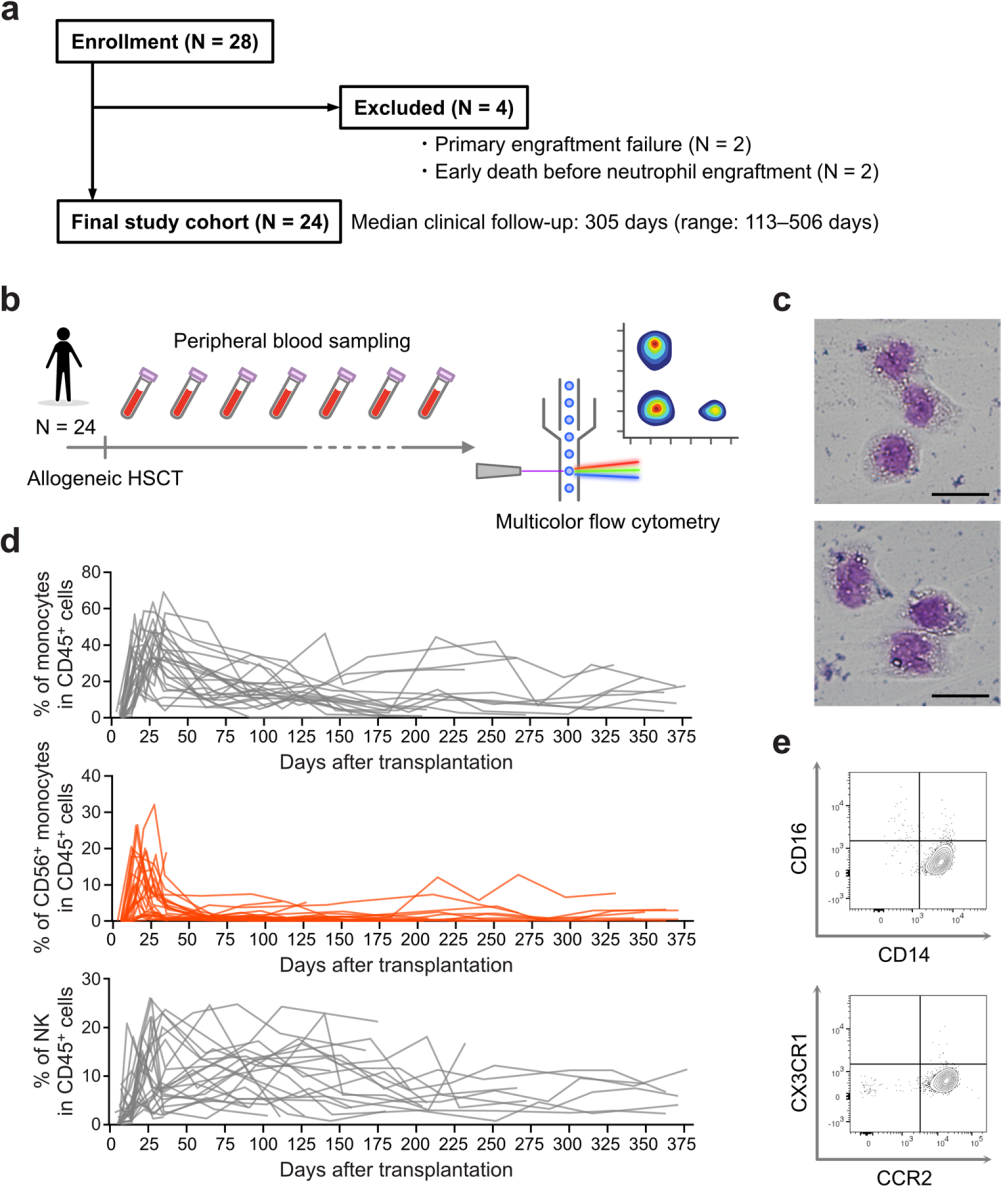
Peripheral blood analyses reveal a transient expansion of CD56^+^ monocytes following neutrophil engraftment (**a**) Flow diagram of patient enrollment, exclusion, and follow-up. (**b**) Schematic overview of the experimental design. Peripheral blood was collected weekly for the first month after hematopoietic stem cell transplantation (HSCT) and monthly thereafter for up to one year post-transplantation (N = 24). Multicolor flow cytometry was performed on each sample after PBMC isolation. (**c**) May–Giemsa staining of CD45^+^CD3^−^CD19^−^CD11b^+^CD14^+^CD56^+^ cells. The scale bars represent 20 μm. (**d**) Line graphs showing the sequential changes in the percentages of total monocytes, CD56⁺ monocytes, and natural killer (NK) cells in CD45⁺ cells, respectively. (**e**) Representative contour plots showing CD14 vs. CD16 and CCR2 vs. CX3CR1 expression in CD56⁺ monocytes

Since the gene expression profile alone was insufficient to clearly distinguish whether these cells were of the monocyte or NK cell lineage, the dynamics of peripheral monocytes and NK cells, as well as their CD56 expression, were analyzed using FCM in patients who were treated with allogeneic HSCT. Twenty-eight patients were initially enrolled in the study; however, four cases were excluded from the final analysis due to an inability to assess post-transplant cellular kinetics resulting from primary engraftment failure or early death before neutrophil engraftment (Fig. 2a). A total of 24 patients were analyzed by prospective monitoring of peripheral blood cells. The patient characteristics and clinical outcomes are summarized in Table 1 and Table S1, respectively. PBMCs were collected weekly for the first month after HSCT and monthly thereafter for up to one year post-transplantation (Fig. 2b). May–Giemsa staining was used to confirm the morphological characteristics of the cells of interest, identifying CD45^+^CD3^−^CD19^−^CD11b^+^CD14^+^CD56^+^ cells as monocytes (Fig. 2c). Although no consistent kinetics were observed in the percentage of total monocytes or NK cells among the CD45^+^ cells, a transient increase in the percentage of CD56⁺ monocytes was notably observed shortly after neutrophil engraftment (Fig. 2d). The percentage of CD56^+^ cells among the monocytes exhibited comparable kinetics (Fig. S3). This fraction was confirmed as a classical monocyte exhibiting CD14^+^CD16^−^ and CCR2^+^CX3CR1^−^ phenotypes. (Fig. 2e).

**Table 1:** Patient characteristics

	*N* = 24	%
Age, median (range) [yr]	41.5 (17-61)	
Sex		
Female	10	41.7
Male	14	58.3
Diagnosis		
MDS	3	12.5
AML	11	45.8
ALL	9	37.5
MPAL	1	4.2
HCT-CI		
0	18	75.0
1	4	16.7
≥ 2	2	8.3
Stem cell source		
BM	9	37.5
PB	8	33.3
CB	7	29.2
HLA disparity		
8/8 matched BM/PB	16	66.7
7/8 matched BM/PB	1	4.2
≥ 4/6 matched CB	7	29.2
Donor-patient sex		
Female to male	6	25.0
Others	18	75.0
Conditioning regimen		
MAC	23	95.8
NMA/RIC	1	4.2
GVHD prophylaxis		
Tac + MTX	20	83.3
Tac + MTX + ATG	4	16.7

*MDS*: myelodysplastic syndrome; *AML*: acute myeloid leukemia; *ALL*: acute lymphoblastic leukemia; *MPAL*: mixed phenotypeacute leukemia; *HCT-CI*: hematopoietic cell transplantation specific comorbidity index; *BM*: bone marrow; PB: peripheral blood stem cells; *CB*: cord blood; *HLA*: human leukocyte antigen; *MAC*: myeloablative conditioning; *NMA*: non-myeloablative conditioning; *RIC*: reduced-intensity conditioning; *GVHD*: graft-versus-host disease; *Tac*: tacrolimus; *MTX*: methotrexate; *ATG*: anti-thymocyte globulin

### CD56-positive monocytes exhibit a pro-inflammatory gene signature

**Fig. 3:**
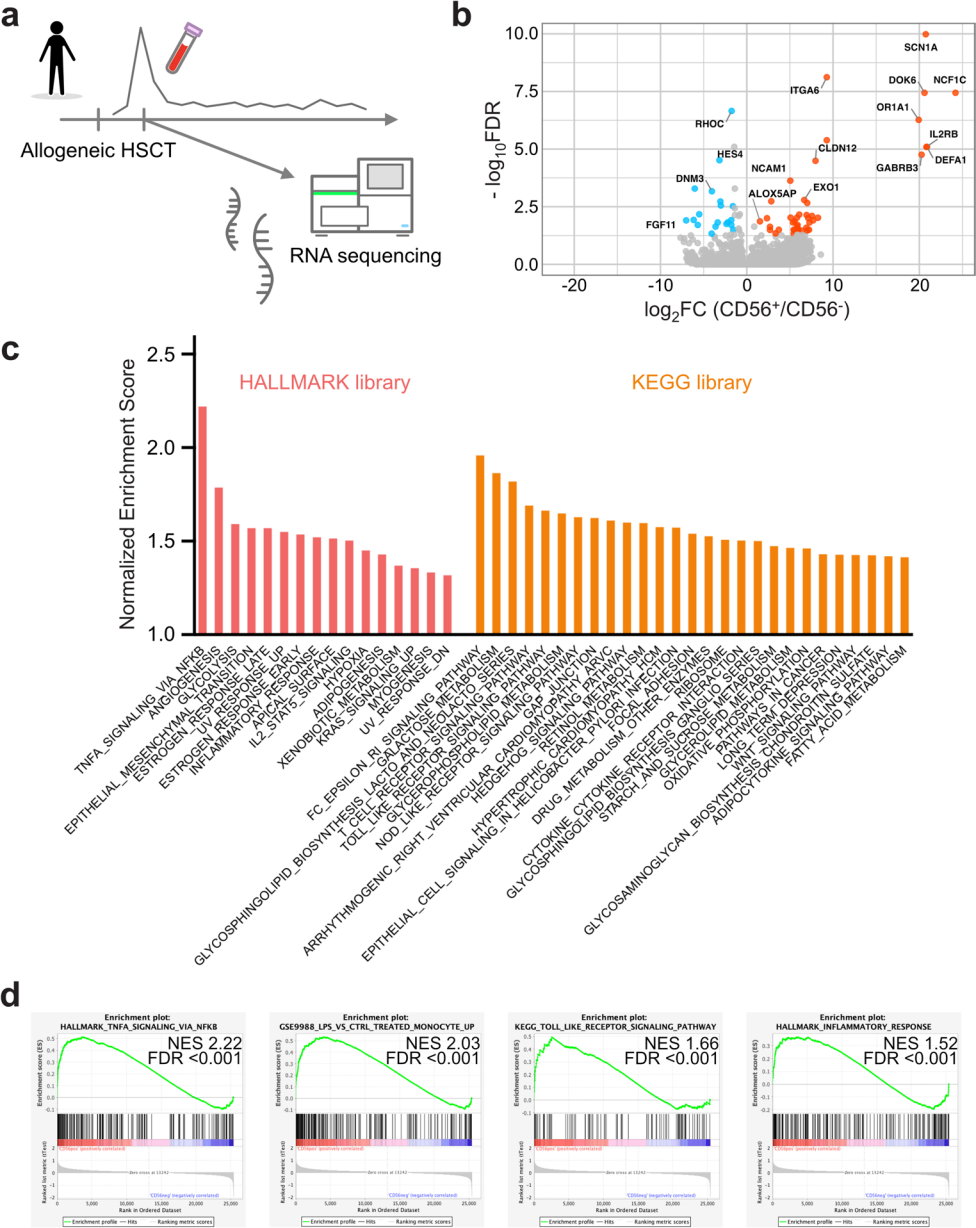
Transcriptomic analysis reveals a pro-inflammatory signature in CD56^+^ monocytes **(a)** Experimental scheme of the RNA sequencing experiment. CD56^+^ and CD56^−^ monocyte fractions were sorted from peripheral blood within 1 week after neutrophil engraftment and subjected to RNA sequencing. **(b)** Volcano plot of differentially expressed genes between the CD56^+^ and CD56^−^ monocytes. **(c)** Bar plot showing normalized enrichment scores of gene sets enriched in CD56^+^ monocytes. Gene sets were derived from the HALLMARK (red) and Kyoto Encyclopedia of Genes and Genomes (KEGG; orange) libraries. **(d)** GSEA plots for selected gene sets significantly enriched in CD56^+^ monocytes, including TNFα signaling, LPS-treated monocyte, Toll-like receptor signaling, and inflammatory response gene set for the CD56^+^ monocytes compared with the CD56^−^ monocytes. NES, normalized enrichment score; FDR, false discovery rate

Because CD56^+^ monocytes in the peripheral blood are a minor cell population, functional evaluation of this rare population is challenging. Therefore, their molecular characteristics were analyzed through transcriptome analysis. RNA sequencing was performed on sorted CD56^+^ and CD56^−^ monocytes, which were collected from an independent cohort within one week of neutrophil engraftment (Fig. 3a; *N* = 4). Cell sorting was performed prior to the onset of acute GVHD achieving high purity (> 96%) in all four patients (Table S4 and S5). Differential gene expression analysis between CD56⁺ and CD56⁻ monocytes identified unique expression genes in the CD56⁺ subset (Fig. 3b). As expected, *NCAM1* was expressed at significantly higher levels in CD56^+^ monocytes than in CD56^−^ monocytes (log_2_[fold change] 5.04, -log_10_[false discovery rate] 3.63). Additionally, the upregulation of genes such as integrin subunit alpha 6 (*ITGA6*), interleukin 2 receptor subunit beta (*IL2RB*), and arachidonate 5-lipoxygenase activating protein (*ALOX5AP*) in CD56⁺ monocytes suggested an association with enhanced cell migration and pro-inflammatory functions [25, 26]. GSEA revealed that CD56⁺ monocytes were significantly enriched for pro-inflammatory gene signatures, including ‘tumor necrosis factor (TNF) α signaling’, ‘inflammatory response’, ‘lipopolysaccharide (LPS)-treated monocyte’, and ‘Toll-like receptor signaling’ compared to CD56⁻ monocytes (Fig. 3c and d). Collectively, these findings suggest that CD56⁺ monocytes exhibit a pro-inflammatory phenotype and may reflect the allogeneic immune responses following allogeneic HSCT.

### Reduced frequency of CD56⁺ monocytes at neutrophil engraftment is associated with a lower incidence of acute GVHD

**Fig. 4:**
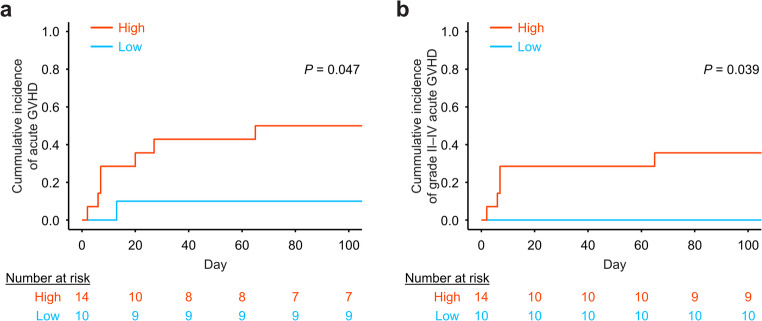
Reduced frequency of CD56⁺ monocytes is associated with a lower incidence of acute GVHD **(a)** Cumulative incidence of acute GVHD in patients stratified according to high and low percentages of CD56^+^ monocytes. **(b)** Cumulative incidence of grade II–IV acute GVHD in the same groups. These analyses were performed using the timepoint of CD56^+^ monocyte assessment at neutrophil engraftment as the landmark, defined as day 0 on the x-axis

Finally, we assessed whether the kinetics of CD56⁺ monocytes were associated with the clinical outcomes following allogeneic HSCT. As CD56^+^ monocyte expansion was a post-transplant event, a landmark at neutrophil engraftment was set, which is a clinically less diverse time point, and before the onset of acute GVHD. Based on ROC curve analysis, we determined that a CD56^+^ monocyte percentage of 6.3% could be used as a cutoff value for predicting subsequent acute GVHD development (area under the curve = 0.65, 95% confidence interval: 0.42–0.88). Patients with a lower percentage of CD56⁺ monocytes had a lower incidence of subsequent acute GVHD than those with a higher percentage (Fig. 4a). This trend remained consistent when the analysis was restricted to patients with grades II–IV acute GVHD (Fig. 4b). Cord blood transplantation was significantly more prevalent in the low CD56^+^ monocyte group when comparing the clinical backgrounds of the high and low CD56^+^ monocyte groups (Table 2). Although no significant difference in the cumulative incidence of acute GVHD was detected among different graft sources, a correlation was observed between donor age and the percentage of CD56⁺ monocytes (*r* = 0.67, 95% confidence interval: 0.36–0.85, *P* = 0.0003; Fig. S4a–c). We found no significant association with other clinical background shown in Table 1 and the percentage of CD56^+^ monocyte at neutrophil engraftment. Additionally, no apparent association was observed between the percentage of CD56⁺ monocytes and affected organs or their clinical scores in acute GVHD (Table S6). The median time from the assessment of the CD56⁺ monocyte percentage at neutrophil engraftment to the onset of acute GVHD was 10 days (range, 2–65 days).

**Table 2:** Patient characteristics according to CD56^+^ monocytes (high vs. low)

	Low (*N* = 10)	High (*N* = 14)	*P* value
Age, median (range) [yr]	45.5 (17-61)	37 (19-60)	0.87^†^
Sex, N (%)			1.00^♭^
Female	4 (40)	6 (42.9)	
Male	6 (60)	8 (57.1)	
Diagnosis, N (%)			0.86^♭^
MDS	0 (0)	3 (21.4)	
AML	7 (70)	4 (28.6)	
ALL	3 (30)	6 (42.9)	
MPAL	0 (0)	1 (7.1)	
HCT-CI, N (%)			1.00^♭^
0	7 (70)	11 (78.6)	
1	2 (20)	2 (14.3)	
≥ 2	1 (10)	1 (7.1)	
Stem cell source, N (%)			0.03^♭^
BM	2 (20)	7 (50)	
PB	2 (20)	6 (42.9)	
CB	6 (60)	1 (7.1)	
HLA disparity, N (%)			0.02^♭^
8/8 matched BM/PB	4 (40)	12 (85.7)	
7/8 matched BM/PB	0 (0)	1 (7.1)	
≥ 4/6 matched CB	6 (60)	1 (7.1)	
Donor-patient sex, N (%)			0.19^♭^
Female to male	4 (40)	2 (14.3)	
Others	6 (60)	12 (85.7)	
Conditioning regimen, N (%)			0.42^♭^
MAC	9 (90)	14 (100)	
NMA/RIC	1 (10)	0 (0)	
GVHD prophylaxis, N (%)			0.11^♭^
Tac + MTX	10 (100)	10 (71.4)	
Tac + MTX + ATG	0 (0)	4 (28.6)	

^†^ Mann–Whitney *U* test, ^♭^ Fisher’s exact test

*MDS*: myelodysplastic syndrome; *AML*: acute myeloid leukemia; *ALL*: acute lymphoblastic leukemia; *MPAL*: mixed phenotypeacute leukemia; *HCT-CI*: hematopoietic cell transplantation specific comorbidity index; *BM*: bone marrow; PB: peripheral blood stem cells; *CB*: cord blood; *HLA*: human leukocyte antigen; *MAC*: myeloablative conditioning; *NMA*: non-myeloablative conditioning; *RIC*: reduced-intensity conditioning; *GVHD*: graft-versus-host disease; *Tac*: tacrolimus; *MTX*: methotrexate; *ATG*: anti-thymocyte globulin

In summary, these results suggest that the percentage of CD56⁺ monocytes at neutrophil engraftment may serve as an early predictive marker for acute GVHD development.

## Discussion

Despite the recent advances in therapeutic drugs for allogeneic HSCT and its several complications, more than half of patients continue to experience severe complications, including acute GVHD. Extensive efforts have been made to develop clinical predictive markers for acute GVHD. However, no definitive markers have yet been established. Immune cell-based markers are considered to be more relevant than indirect serum markers, but comprehensive analyses of diverse immune cells after allogeneic HSCT have been limited. Based on scRNA-seq analysis of PBMCs from patients with acute GVHD, this study focused on the kinetics of CD56^+^ monocytes following allogeneic HSCT. A marked increase in the CD56-positive fraction of monocytes was observed early after neutrophil engraftment, preceding the onset of acute GVHD. To the best of our knowledge, this study is the first to describe CD56 expression in monocytes in the context of allogeneic HSCT and its association with acute GVHD using clinical specimens.

CD56, a cell-surface molecule containing five extracellular immunoglobulin-like domains and two fibronectin type III repeats, is alternatively known as ‘NCAM’. It has been well characterized in neurons, where it mediates neural cell adhesion and synaptic plasticity [27]. CD56 is also known to be expressed on immune cells and is widely used as a phenotypic marker for human NK cells and NKT cells [10, 28]. Although CD56 expression is thought to be associated with cell migration, cytotoxic function, and cytokine secretion, the functional role of CD56 expression remains to be elucidated [29]. Recent studies have identified unique CD56-expressing monocyte fractions in various inflammatory diseases and malignancies, highlighting them as a distinct monocyte subset [10]. A monocyte fraction with weak CD56 expression was initially identified in healthy individuals, and shortly after that, it was reported that the percentage of CD14⁺CD56⁺ monocytes was 2.7 times higher in patients with active Crohn’s disease than in healthy controls [30, 31]. Similarly, increase in CD56⁺ monocytes has been observed in rheumatoid arthritis [32]. Furthermore, an increased frequency of CD56⁺ monocytes has been reported in COVID-19 patients, correlating with disease severity [33, 34]. Experimental study revealed that CD56⁺ monocytes produced higher levels of TNF and generated greater amounts of reactive oxygen species than CD56⁻ monocytes upon stimulation with LPS [32]. Inappropriate immune activation, often triggered by endothelial dysfunctions, is a crucial factor in the development of acute GVHD [35, 36]. In addition, LPS/Toll-like receptor 4 signaling also plays a key role in the pathogenesis of acute GVHD [37–39]. The pro-inflammatory phenotype and high sensitivity to LPS observed in CD56^+^ monocytes may reflect their role as effective antigen-presenting cells in acute GVHD development. In fact, an in vitro experiment has demonstrated that co-culture with CD56^+^ monocytes significantly enhanced the proliferation of HLA-mismatched allogeneic T cells [30].

Transcriptome analysis in the current study revealed an enhanced inflammatory gene signature in CD56^+^ monocytes isolated early after neutrophil engraftment compared to their CD56⁻ counterparts. These findings support the hypothesis that CD56⁺ monocytes may contribute to inflammatory complications such as acute GVHD after allogeneic HSCT. In addition, the percentage of CD56^+^ monocytes at neutrophil engraftment clearly stratified the risk of acute GVHD. Notably, the expansion of CD56⁺ monocytes was less evident after cord blood transplantation. Age-associated hematopoietic changes are characterized by reduced lymphopoiesis, red blood cell abnormalities, and a shift toward myeloid lineage differentiation [40, 41]. These ‘myeloid-biased’ hematopoietic stem cells (my-HSCs) give rise to pro-inflammatory myeloid cells and are increasingly recognized as therapeutic targets for age-related diseases [40]. In allogeneic HSCT, increased donor age has been associated with higher transplant-related mortality [42]. The attenuated expansion of CD56⁺ monocytes in cord blood recipients might reflect more balanced hematopoiesis and could contribute to the lower incidence of acute GVHD.

Several limitations of this study must be acknowledged. Notably, functional evaluation of CD56 expression in monocytes was not feasible owing to the limited number of CD56^+^ monocytes available, especially early after the neutrophil engraftment. Further technological advances are required to elucidate the physiological role of CD56 expression in monocytes after allogeneic HSCT. In addition, the small size of our cohort may have limited the statistical power of the ROC analysis. Future validations in larger, more diverse patient cohorts would be highly valuable to confirm the clinical utility of the identified CD56^+^ monocyte cutoff value in predicting acute GVHD. Furthermore, the reduced sampling frequency in the later phase after HSCT was another limiting factor in this study. This was primarily due to the transition to outpatient care. This constrained the timely evaluation of CD56^+^ monocytes in relation to chronic GVHD and other late post-transplant complications. Future studies examining CD56^+^ monocyte kinetics before and after active chronic GVHD treatment could provide new insights from a different clinical perspective.

In conclusion, this study identified a distinct and transient increase in CD56^+^ monocytes following allogeneic HSCT, along with their pro-inflammatory signatures. A lower frequency of CD56⁺ monocytes was associated with reduced incidence of acute GVHD, suggesting their utility as a promising early cellular biomarker for predicting acute GVHD and highlighting their potential as therapeutic targets.

## Supplementary Information

Below is the link to the electronic supplementary material.

Supplementary Material 1

Supplementary Material 2

## Data Availability

The raw count data from this study have been deposited in NCBI’s Gene Expression Omnibus and are accessible through accession number GSE300085.
