# Supplementary material for: Percentage of CD56+ monocytes at neutrophil engraftment is associated with the incidence of acute graft-versus-host disease
Source: Ann Hematol. 2026 Mar 2;105(4):153. doi: 10.1007/s00277-026-06897-2 (PMC12953352; doi:10.1007/s00277-026-06897-2)
Supplement: Supplementary file 1 — Supplementary Material 1 [file 277_2026_6897_MOESM1_ESM.pdf]

Supplementary Figures S1–4

Article title:	Percentage of CD56$^+$ monocytes at neutrophil engraftment is associated with the incidence of acute graft-versus-host disease
Journal name:	*Annals of Hematology*
Author names:	Ken Hashimoto[1,2], Takahiko Sato[1,2], Yuichi Ishikawa[1], Yuki Okuhiro[2], Daisuke Sugiyama[2], He Zhang[2], Sachiko Ito[2], Yuichiro Inagaki[3], Kotaro Miyao[3], Masashi Sawa[3], Takanobu Morishita[4,5], Tatsunori Goto[4], Tetsuya Nishida[4], Nobuaki Fukushima[6], Kazutaka Ozeki[6], Ryo Hanajiri[1], Seitaro Terakura[1], Hiroyoshi Nishikawa[2,7,8,9], Hitoshi Kiyoi[1]
Affiliations:	[1] Department of Hematology and Oncology, Nagoya University Graduate School of Medicine, Nagoya, Japan.<br>[2] Department of Immunology, Nagoya University Graduate School of Medicine, Nagoya, Japan.<br>[3] Department of Hematology and Oncology, Anjo Kosei Hospital, Anjo, Japan<br>[4] Department of Hematology, Japanese Red Cross Aichi Medical Center Nagoya Daiichi Hospital, Nagoya, Japan<br>[5] Department of Blood and Marrow Transplantation & Cellular Therapy, Fujita Health University, Toyoake, Japan<br>[6] Department of Hematology and Oncology, Konan Kosei Hospital, Konan, Japan.<br>[7] Division of Cancer Immunology, Research Institute / Exploratory Oncology Research & Clinical Trial Center (EPOC), National Cancer Center, Tokyo/Chiba, Japan.<br>[8] Division of Cancer Immune Multicellular System Regulation, Center for Cancer Immunotherapy and Immunobiology, Kyoto University Graduate School of Medicine, Kyoto, Japan<br>[9] Kindai University Faculty of Medicine, Osaka-sayama, Japan.
Lead contact:	Takahiko Sato
E-mail:	t.sato@med.nagoya-u.ac.jp

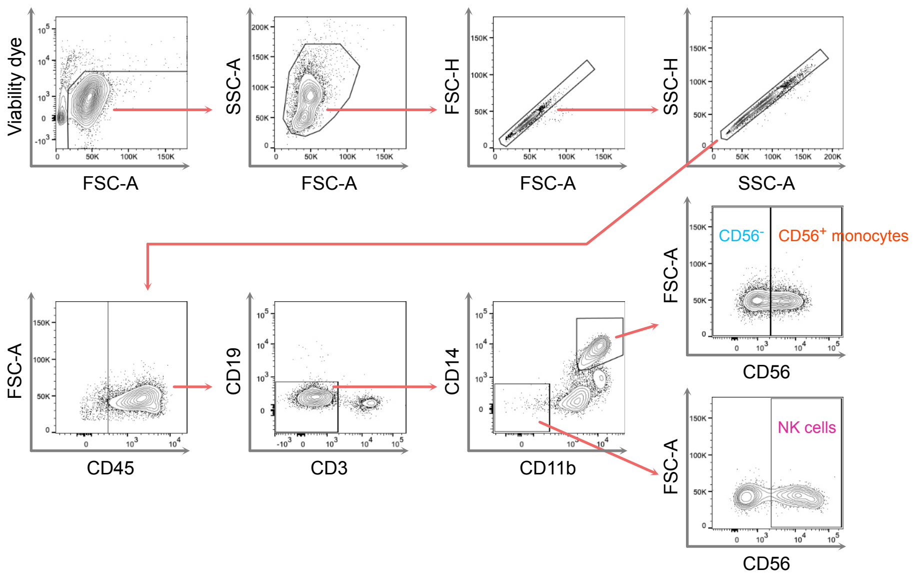

Figure S1. Gating strategies for monocytes and NK cells

Flow cytometric gating scheme used to define the CD56^{+} monocyte population

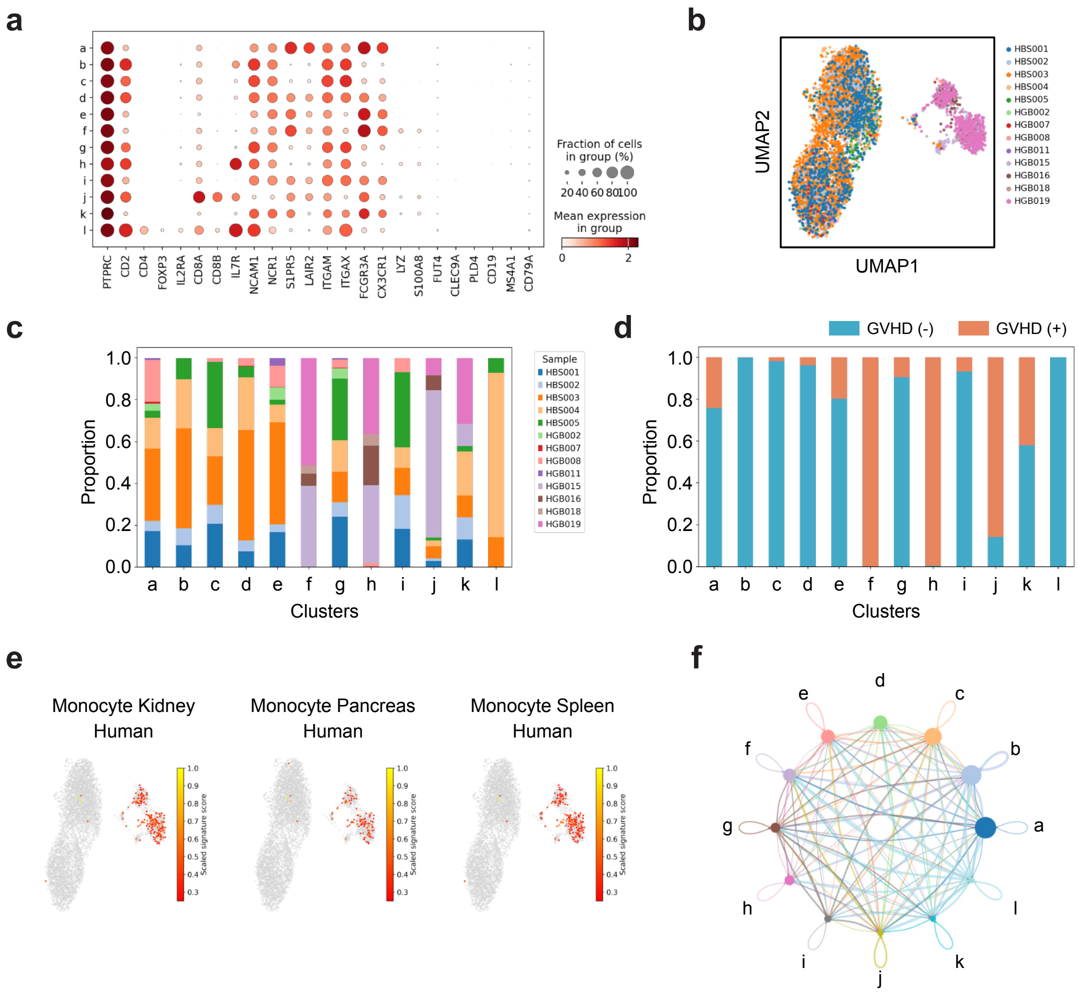

Figure S2. Characteristics of cell clusters identified by scRNA-seq analysis

(a) Dot plot depicting the percentage of cells and mean expression levels of representative marker genes in each cluster. **(b)** UMAP visualization based on clinical sample annotations. **(c)** Bar plot showing the proportion of individual samples within each cluster. **(d)** Bar plot showing the proportion of acute GVHD(+) cases in each cluster. **(e)** Expression intensities of the top three monocyte-associated gene sets visualized over the UMAP plot. **(f)** Predicted cell-to-cell interaction networks across cell clusters were estimated using the CellChat package.

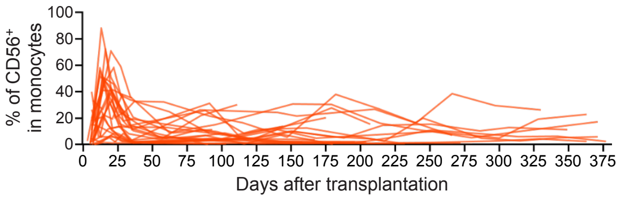

Figure S3. CD56^{+} monocytes expand early after allogeneic HSCT

Line graph showing the sequential changes in the percentages of CD56^{+} cells in total monocytes.

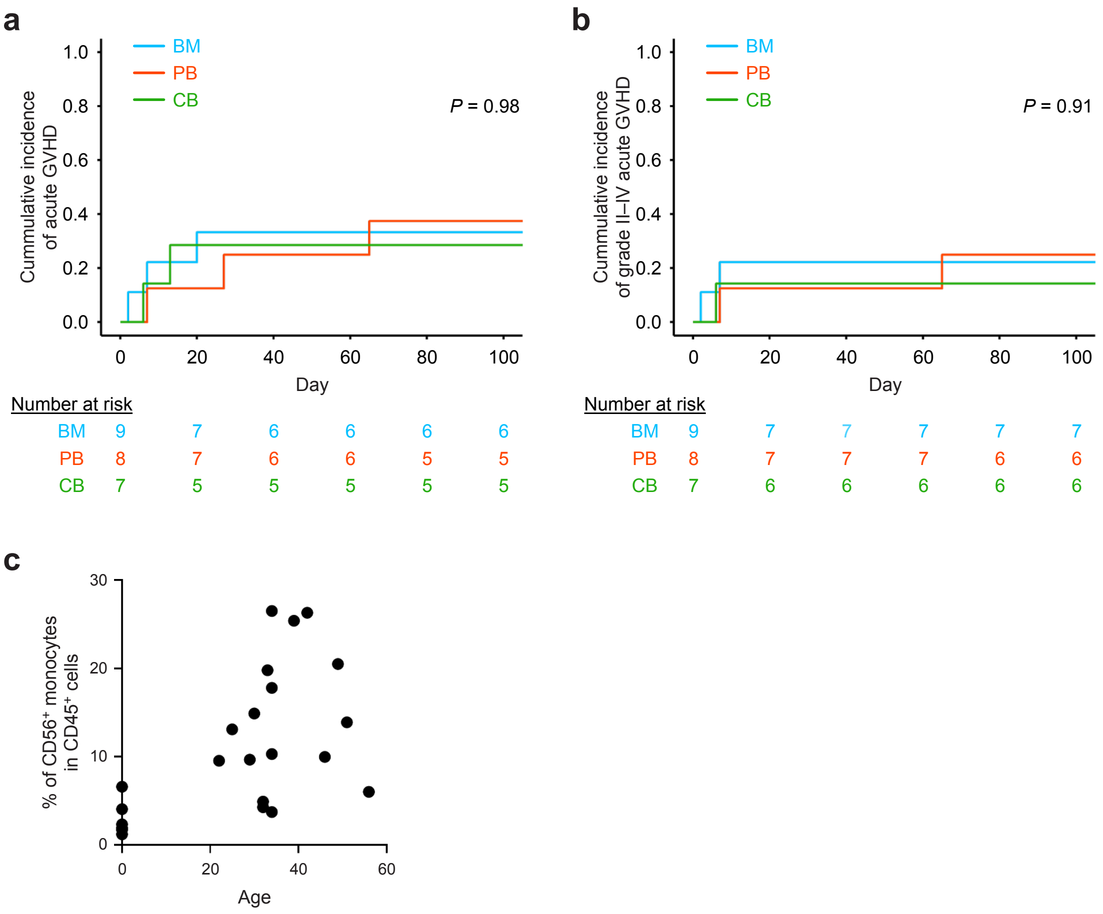

Figure S4. Impact of graft source on acute GVHD and donor age in relation to CD56$^+$ monocyte percentage

(a) Cumulative incidence of acute GVHD according to stem cell sources. **(b)** Cumulative incidence of grade II–IV acute GVHD according to stem cell sources. **(c)** Scatter plot demonstrating the correlation between donor age and the CD56$^+$ monocyte percentage among CD45$^+$ peripheral blood cells at neutrophil engraftment.
